# Earliness and morphotypes of common wheat cultivars of Western and Eastern Siberia

**DOI:** 10.18699/VJGB-22-81

**Published:** 2022-11

**Authors:** S.E. Smolenskaya, V.М. Efimov, Yu.V. Kruchinina, B.F. Nemtsev, G.Yu. Chepurnov, Е.S. Ovchinnikova, I.А. Belan, Е.V. Zuev, Zhou Chenxi, V.V. Piskarev, N.P. Goncharov

**Affiliations:** Institute of Cytology and Genetics of the Siberian Branch of the Russian Academy of Sciences, Novosibirsk, Russia; Institute of Cytology and Genetics of the Siberian Branch of the Russian Academy of Sciences, Novosibirsk, Russia Novosibirsk State University, Novosibirsk, Russia; Institute of Cytology and Genetics of the Siberian Branch of the Russian Academy of Sciences, Novosibirsk, Russia; Siberian Research Institute of Plant Production and Breeding – Branch of the Institute of Cytology and Genetics of the Siberian Branch of the Russian Academy of Sciences, Novosibirsk, Russia; Institute of Cytology and Genetics of the Siberian Branch of the Russian Academy of Sciences, Novosibirsk, Russia; Institute of Cytology and Genetics of the Siberian Branch of the Russian Academy of Sciences, Novosibirsk, Russia; Omsk Agrarian Scientific Center, Omsk, Russia; Federal Research Center the N.I. Vavilov All-Russian Institute of Plant Genetic Resources (VIR), St. Petersburg, Russia; Novosibirsk State University, Novosibirsk, Russia; Siberian Research Institute of Plant Production and Breeding – Branch of the Institute of Cytology and Genetics of the Siberian Branch of the Russian Academy of Sciences, Novosibirsk, Russia; Institute of Cytology and Genetics of the Siberian Branch of the Russian Academy of Sciences, Novosibirsk, Russia Novosibirsk State University, Novosibirsk, Russia

**Keywords:** common wheat, Vrn genes, commercial and local cultivars, earliness, morphotype, breeding, мягкая пшеница, гены Vrn, селекционные и местные сорта, скороспелость, морфотип, селекция

## Abstract

The global and local climate changes determine the producing of highly-adaptive common (bread) wheat commercial cultivars of a new generation whose optimal earliness matches the climatic features of the territory where the cultivars are farmed. Principal component analysis involving our own and published data has been applied to investigate 98 commercial common wheat cultivars from Western and Eastern Siberia comparing their morphotypes; cultivar zoning time; length of the vegetation period; 1000-grain weight, and inheritance of spring growth habit. It demonstrated that the dominant Vrn gene polymorphism determining the spring growth habit of the Siberian cultivars was minimally polymorphic. In 75 % of the tested cultivars, the spring growth habit was controlled by digenic, namely dominant Vrn-A1 and Vrn-B1 genes. In 25 % of them (24 cultivars), spring growth habit is controlled by a single gene. In 19 and 5 of these cultivars spring growth habit is controlled by only one dominant gene, Vrn-B1 or Vrn-A1, respectively. In cv. Tulun 15, a trigenic control was identified. A conclusion about the optimality of the digenic control for the climatic conditions of both Western and Eastern Siberia has been confirmed. However, since none of the tested cultivars had the dominant Vrn-D1 gene typical of the regions of China and Central Asia bordering Siberia, it can be considered as an additional argument in favor of the European origin of Siberian common wheat cultivars. The revealed high frequency of the Vrn-B1c allele in the Western Siberian cultivars and the Vrn-B1a allele in the Eastern Siberian cultivars suggests their selectivity. The analysis also confirmed the dominance of red glume (ferrugineum, milturum) and awned spike (ferrugineum, erythrospermum) varieties in the Eastern Siberian cultivars, and white glume and awnedless spike (lutescens and albidum) ones in the Western Siberian cultivars. Small grain size cultivars are more typical of Eastern than Western Siberia. The retrospective analysis based on the cultivars’ zoning time included in the “State Register for Selection Achievements Admitted for Usage” brought us to the conclusion that the earliness/lateness of modern Siberian commercial cultivars was not regionally but rather zonally-associated (taiga, subtaiga, forest-steppe and steppe zones).

## Abstract (ru)

Глобальное и локальное изменение климата обусловливает создание высокоадаптивных сортов яровой мягкой пшеницы нового типа с оптимальной скороспелостью, отражающей региональные особенности климатических условий территорий ее возделывания. Методом главных компонент с использованием собственных данных и результатов, опубликованных другими исследователями, проведено сравнение 98 селекционных коммерческих сортов яровой мягкой пшеницы Западной и Восточной Сибири по морфотипу, времени районирования, длине вегетационного периода, массе 1000 зерен и характеру наследования ярового типа развития. Полиморфизм по доминантным генам Vrn, контролирующим яровость у селекционных сортов Сибири, минимален. У 75 % из них он контролируется дигенно доминантными Vrn-A1 и Vrn-B1; у 25 % – моногенно доминантными генами (у 24 сортов, из них у 19 и 5 соответственно только одним доминантным геном – Vrn-A1 или Vrn-B1). У одного сорта, Тулун 15, описан тригенный контроль. Подтвержден вывод об оптимальности для климатических условий как Западной, так и Восточной Сибири контроля яровости двумя доминантными генами Vrn. Ни у одного из сортов не обнаружено характерного для приграничных с Сибирью областей Китая и Центральной Азии доминантного гена Vrn-D1. Данное наблюдение может служить дополнительным аргументом в пользу гипотезы европейского происхождения сортимента Сибири. Выявлена повышенная частота встречаемости аллеля Vrn-B1с у сортов Западной Сибири и аллеля Vrn-B1а у сортов Восточной Сибири, что позволяет говорить об их селективности. Подтверждено преобладание красноколосых (ferrugineum, milturum) и остистых (ferrugineum, erythrospermum) разновидностей в Восточной Сибири, безостых и белоколосых (lutescens и albidum) – в Западной Сибири. Для Восточной Сибири характерны более мелкозерные сорта. Выполненная ретроспектива по годам районирования (включения в «Государственный реестр селекционных достижений, допущенных к использованию» РФ) позволяет отметить, что скороспелость/позднеспелость современных сортов Сибири не имеет ярко выраженной региональной компоненты, а является зональной (тайга, подтайга, лесостепь, степь).

## Introduction

Wheat is one of the three most wide-spread crops in the world, but, unlike rice and corn, both its winter and spring cultivars are widely cultivated. Spring common (bread) wheat, in this respect, mains a crucial crop for the regions of South and North America, Australia, Central and South- East Asia as well as for those of Northern Asia which have harsh continental climate (Morgounov et al., 2018; Garcia et al., 2019; Rivelli et al., 2021).

In the Russian Federation, these are mainly the winter and spring cultivars of common wheat (Triticum aestivum L.) and the spring cultivars of macaroni ones (T. durum Desf.) that are farmed. Only insignificant areas are taken to grow winter macaroni (Fomenko, Grabovets, 2016) and Indian dwarf wheat (T. sphaerococcum Perciv.) (Bespalova et al., 2015) cultivars as well as emmer (T. dicoccum Schrank. ex Schübler) (Temirbekova et al., 2020). The “State Register for Selection Achievements Admitted for Usage (National List)” also includes two cultivars of Rivet wheat (T. turgidum L.) and one of spelt (T. spelta L.) (State Register…, 2021)1. Among the mentioned ones, the common wheat species the backbone of Russia’s crop farming, dominating not only over other species of genus Triticum L. but overall crops cultivated in the country.

For the last two species, the “State Register…” indicates no type of growth habit (spring vs. winter). Moreover, the State Commission for Breeding Test System and Protection uses the wheat taxon names nonrelated to the Russian scientific tradition (Dorofeev et al., 1979; Goncharov, 2011).

Along with Southern Urals and the Volga region, Eastern and Western Siberia are main territories of spring common wheat farming in Russia. The global and local climate changes determine the producing of highly-adaptive common wheat cultivars of a new generation with optimal earliness to match the climatic features of the territory where they are farmed, including the harsh continental areas of Western and Eastern Siberia.

The duration of vegetation period (earliness) in wheat, as the most important adaptive trait (Lozada et al., 2021), determines not only plant productivity (yield) but also affects its resistivity to such external environmental stress factors as drought, low temperature, insects, diseases, etc. (Zotova et al., 2019). Moreover, farming spring cultivars of different earliness enables one to control harvesting times to reduce peak loads on agricultural machinery and yield losses due to overmature (Belan et al., 2021). Duration of vegetation period in wheat is a complex trait whose extent is mainly determined by the allele diversity of the Vrn genes controlling the type growth habit (spring vs. winter) and response to vernalization and by that of the Ppd genes controlling response to photoperiod (Kiseleva, Salina, 2018). Currently, a set of VRN loci (Vrn-A1, Vrn-B1, Vrn-D1, Vrn-B3, and Vrn-D4 genes) (Goncharov, 2003; Yan et al., 2003, 2004, 2006; Kippes et al., 2014) and at least two PPD loci (Ppd- B1, Ppd-D1 genes) (Welsh et al., 1973; Beales et al., 2007; Diaz et al., 2012) have been identified in hexaploid wheat species.

In wheat, the molecular basis of genetic control of earliness is being intensively investigated (Royo et al., 2020), but there is still considerable uncertainty when it comes to its phenotypical manifestations determined by the interaction of Vrn and Ppd gene alleles. Some experts claim Vrn genes control up to 75 % of the variability related to the duration of the vegetation period (earliness), while Ppd genes account for only 20 % of them (Stelmakh, 1981). However, in the spring cultivars and when winter and spring wheat are cultivated northward of 55° N and southward of 55° S, the influence of these genes on the trait manifestation changes significantly. The results of the correlation analysis of the vegetation period duration in the wheat cultivars with yield have been contradictory (Vedrov, 2006; Meng et al., 2016; Piskarev et al., 2018; Rigin et al., 2018; Sidorov, 2018; Kuz’min et al., 2019; among others) and, due to their importance, call for comprehensive study.

The current diagnostic molecular markers have been developed to identify the alleles of the Vrn and Ppd genes. They have made it possible to detect the presence/absence of dominant Vrn and Ppd genes in both local and commercial (cultivated) wheat cultivars from the countries of Europe, Asia, North and South America, Africa and Australia (Zheng et al., 2013; Gomez et al., 2014; Cho et al., 2015; Shcherban et al., 2015; Whittal et al., 2018; Royo et al., 2020). It has been shown that the most early-maturing cultivars possess at least three dominant Vrn genes (Zhang et al., 2008; Rigin et al., 2019, 2021а, b), among which some authors include the rare dominant Vrn-B3 allele (Zhang et al., 2008). It is noteworthy that this allele has been detected in the only one Russian cv. Tulun 15 being most early maturing among those permitted for cultivate in Siberia (Lysenko et al., 2014). A new dominant Vrn-A3 gene controlling the early maturity in the accession TN26 of T. dicoccum has been described (Nishimura et al., 2018). It is assumed that the early maturity is caused by the GATA-box element found in this gene, while this locus as well as the VRN-2 one described in T. monococcum L. (Tan, Yan, 2016) is not functional in hexaploid wheat cultivars.

Genogeographic studies have been performed in our country to investigate the Vrn genes in spring wheat landraces (Genotypes…, 1985; Goncharov, Shitova, 1999; Moiseeva, Goncharov, 2007) as well as in the Russian commercial cultivars (Genotypes…, 1985; Catalogue…, 1987; Shcherban et al., 2012b; Lysenko et al., 2014; etc.). The other investigated cultivars were spring common wheats from Siberia (Fait, Stelmakh, 1993; Dzhalpakova et al., 1996; Likhenko et al., 2014; etc.); the local cultivars of seven hexaploid wheat species from different regions of Eurasia (Dragovich et al., 2021); macaroni wheat cultivars (Dzhalpakova et al., 1995; Konopatskaia et al., 2016), and emmer landraces (Rigin et al., 1994). As an earliness donor, Aegilops squarrosa L. (syn. Aegilops tauschii Coss.) the D genome donor of polyploid wheats has been suggested (Goncharov, Chikida, 1995).

The search for Vrn gene polymorphisms and studying its influence to the earliness expression has been one of the key directions of the Russian wheat breeding programs, including Eastern and Western Siberia since dominant Ppd genes are not that widespread in Siberian cultivars (Likhenko et al., 2014; Balashova, Fait, 2021).

The purpose of the present study was to compare the bred (commercial) cultivars of spring common wheat from Western and Eastern Siberia for their dominant Vrn alleles and morphotypes to investigate their effect on the earliness, yield and cultivar zoning time.

## Materials and methods

Biological material. Only cultivars of common wheat included in the “State Register for Selection Achievements Admitted for Usage (National List)” (State Register…, 2021) were studied in our investigation. Information on inheritance of them spring growth habit included in the was either obtained in this investigation or taken from publications (Dzhalpakova et al., 1996; Likhenko et al., 2014; Lysenko et al., 2014, etc.) (Supplementary Material)2. The data about morphotypes, duration of vegetation period and 1000-grain weight were taken from the official publications of The State Commission for Breeding Test System and Protection (Guidelines…, 1928, 1937; among others) ,(As it has been mentioned earlier, the vegetation period grades of the different cultivars studied in different sites almost never change (Goncharov, Efimov, 1990; Smiryaev et al., 1992). For that reason, in our study we followed E.S. Kuznetsova’s (1929) approach who considered that studying a cultivar core collection set enabled one to obtain proper information about the whole species polymorphism). “Catalog…” (2009), since they provided information on trait fluctuations for all state agricultural facilities in a region, and these data were necessary for obtaining integral estimations. In total, the information about 98 commercial spring common wheat cultivars from Eastern and Western Siberia (zoning time from 1929 through 2021) was collected as well as the information concerning four our breeding lines was used (see Supplementary Material).

Supplementary Materials are available in the online version of the paper: http://vavilov.elpub.ru/jour/manager/files/Suppl_Smolenskaya_27_7.pdf


In case of incomplete data, a cultivar was removed from analysis, such as the cvs Soanovskaya 4 and Khludovka. In addition, the cultivars produced in Siberian agricultural institutions but zoned in other regions such as Perm (cv. Tayezhnaya) or the Far East (cv. Priobskaya) were removed. Since the local cultivar data were mainly represented by East Siberian cultivars (Goncharov, Shitova, 1999) and Tuva landraces (Moiseeva, Goncharov, 2007), they were used only in discussion.

PCR amplification conditions and total DNA isolation protocol. The total DNA isolation and PCR amplification conditions were carried out as described in (Konopatskaia et al., 2016). For PCR amplification were used the primers specific for the Vrn-A1, Vrn-B1 and Vrn-D1 genes (Konopatskaia et al., 2016).

Data. The information about the genotypes and phenotypes of on such biological traits as botanical varieties, the duration of vegetation period (earliness), 1000-grain weight and Vrn gene alleles presented in Supplementary Material. Note that the cultivar botanical varieties were first studied as a whole, e. g., ferrugineum and lutescens, and then subset into their element traits such as spike color, awnedness/awnedlessness, etc. according their spike and grain traits (Table 1).

**Table 1.**
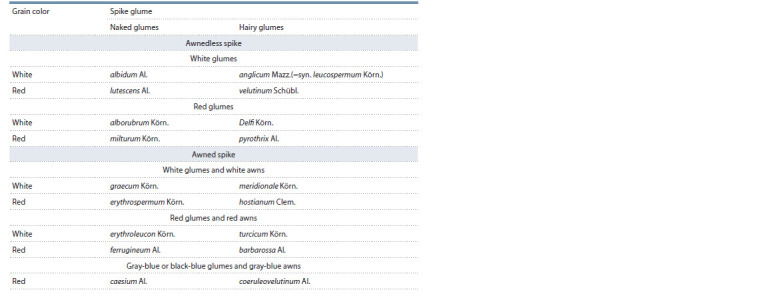
Reduced classification of the most important common wheat botanical varieties (from: Plotnikov et al., 1937)

Statistical analysis. For statistical processing, quantitative and qualitative characteristics of cultivars were used. Both quantitative (mean duration of vegetation period, 1000-grain weight) and binary traits (awnedness/awnedlessness, spike and grain color, Vrn-A1 gene alleles) were aligned and normalized in a way the sum of squares for each of them was equal to one. Every qualitative character, whose gradation exceeded three (Vrn-B1 gene alleles, morphotypes), was coded using a binary number in which one marked belonging to this particular gradation, and zero – to all other graduations used. Since this population still represented just a single trait, it was aligned and normalized for its sum of squares to be equal to one as well. In such a way all the traits were equally weighted. To estimate the principal components for all the investigated biological traits, a Euclidean distance matrix was built and the principal coordinate method was applied (Gower, 1966).

## Results

**Fig. 1.**
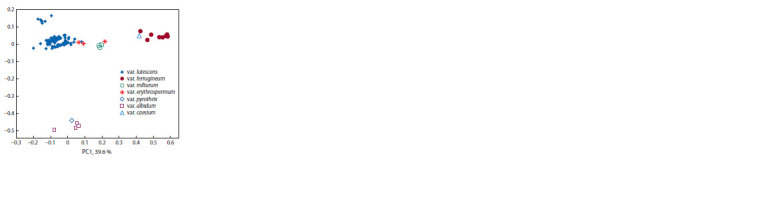
Disposition of cultivar varieties with respect to the f irst two principal components

**Fig. 2.**
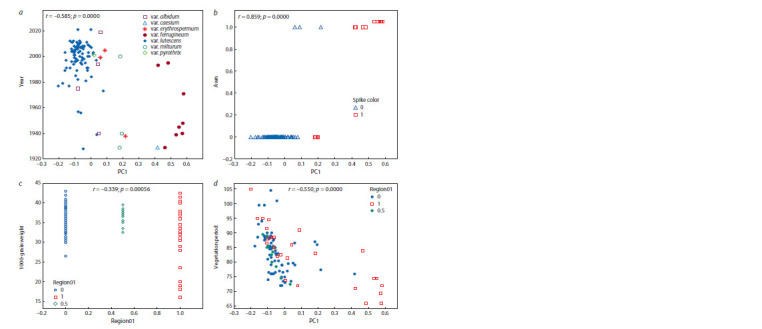
Trait-pair scattering diagram. а – morphotype-based cultivar zoning time against PC1; b – spike color and awnedness against PC1; c – region-based against 1000-grain weight; d – region-based duration of vegetation period against PC1.

**Fig. 3.**
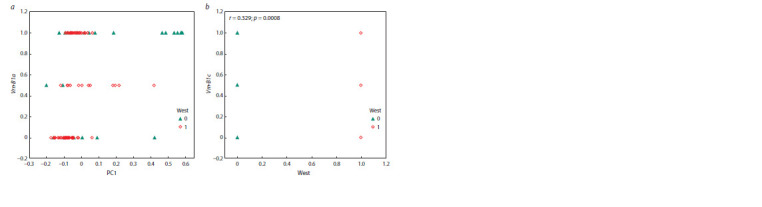
Scatterplot of Vrn-B1а allele and Vrn-B1c allele against: (а) – principal component (PC1); (b) – zoning.

The results of the statistical data processing for the cultivars biological traits and their agronomical characteristics (see Materials and methods) can be seen in Tables 2 and 3, Fig. 1–3. The contributions of the first three principal components gave 69.3 % in total (see Table 2) that comprised around 70 % of the total dispersion. A cultivar agronomical characteristics are included its farming region and the zoning time, i. e., the year it was included in “State Register for Selection Achievements Admitted for Usage (National List)”. In our study, these characteristics were considered as external ones and for that reason were excluded from the principal component estimation. For biological interpretation of the obtained components, it was enough to calculate their correlations with any qualitative traits including biological and agronomical ones (see Table 3).

**Table 2.**
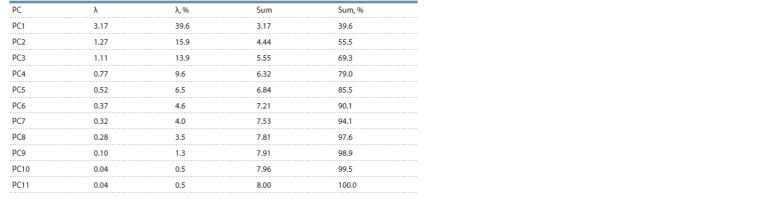
Principal component dispersions (λ) and their accumulated sums (Sum) Notе. РС – the principal components.

**Table 3.**
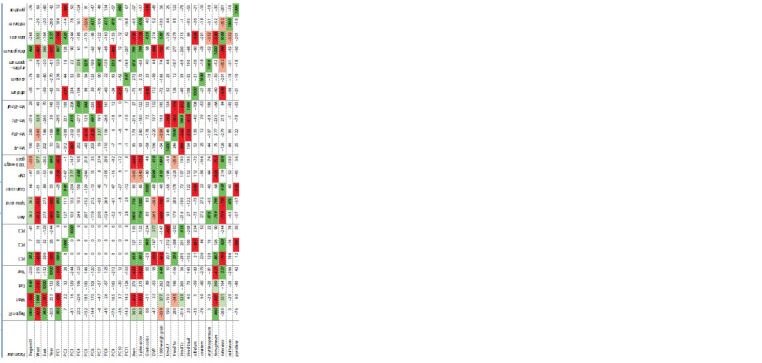
Principal component correlation matrix (×1000) between the biological traits and agronomical characteristics of the studied cultivars Notе. The light-red and light-green colors mark p < 0.001, and red and green – p <10–4; West – Western Siberia, East – Eastern Siberia; PC – the principal component; DVP – duration of vegetation period.

Cultivar position determined, first-hand, by the their morphotypes, namely, lutescens, erythrospermum and milturum varieties take the upper left-hand (LEM group), morphotypes ferrugineum and caesium (FC group) – the upper right-hand, and albidum and pyrothrix (AP group) – lower left-hand corner. Thus, the primary component is determined by the differences between groups LEM and FC, and the secondary – between groups LEM and AP (see Fig. 1).

The agronomical characteristics farming region and cultivar zoning time correlated only with the PC1, giving r = 0.392 and r = –0.585, respectively. The biological traits correlating with the PC1 included the awnedness, spike color, duration of vegetation period, 1000-grain weight, Vrn-B1a allele presence, var. ferrugineum and lutescens. A direct correlation was discovered between Vrn-B1a and 1000-grain weight (r = –0.354), i. e., the presence of this dominant allele resulted in 1000-grain weight reduction. At the same time, the presence of any dominant Vrn gene alleles did not affect the other biological traits. In other words, they correlated only against one another, forming a separated group of principal components from PC3 to PC6.

Hence, the biological meaning of the PC1 (see Fig. 2 and 3) is the early cultivar zoning time, spike awnedness and color of the cultivars from Eastern Siberia as well as their reduced duration of vegetation period, 1000-grain weight and var. lutescens, and increased Vrn-B1a allele frequency and var. ferrugineum.

At the same time, small-grained cultivars are typical for Eastern Siberia than for Western Siberia (r = –0.262), while large-grained cultivars are typical for Western Siberia (r = 0.377) (see Table 3, Fig. 2, c).

Cultivar zoning time. As Fig. 2, a demonstrates, before the 1990th, breeding in Eastern Siberia mainly produced awned varieties, but this tendency has changed since then. Hard to say why.

Vrn genes. An important consideration of our study was the presence/absence in the tested cultivars of VRN-1 locus multiple alleles that control the duration of vegetation period. An important in the study was the identification of alleles of the VRN-1 locus and the assessment of their influence on duration of vegetation period. The polymorphism of the dominant Vrn genes controlling cultivar spring growth habit in the 98 investigated cultivars was minimally expressed (see Supplementary Material). It demonstrated that the dominant Vrn gene polymorphism determining the spring growth habit of the Siberian cultivars was minimally polymorphic. In 75 % of the tested cultivars, the spring growth habit was controlled by digenic, namely dominant Vrn-A1 and Vrn-B1 genes. In 25 % of them (24 cultivars), the spring growth habit of control is monogenic. In 19 and 5 of these cultivars spring growth habit is controlled by only one dominant gene Vrn-A1, Vrn-B1, respectively. In cv. Tulun 15, a trigenic control was identified. A conclusion about the optimality of the digenic control the climatic conditions of both Western and Eastern Siberia has been confirmed. However, since none of the tested cultivars had the dominant Vrn-D1 gene typical for the regions of China and Central Asia bordering Siberia, it can be considered as an additional argument in favor of the European origin of Siberian common wheat cultivars. Two alleles of the dominant Vrn-A1 gene were detected. While the frequency of the Vrn-A1b allele comprised less than 2 %, the Vrn-A1a allele presented in the most of the tested cultivars. The exception is a number of cultivars of Omsk breeding, such as cvs Omskaya 9, Omskaya 12, etc., the spring growth habit of which is determined by the monogenic by dominant gene Vrn-B1, but their number does not exceed 5 % of the assortment (see Supplementary Material).

The Vrn-B1 gene has three alleles, namely the dominant Vrn-B1a, Vrn-B1c alleles and recessive vrn-B1 allele, i. e. neither Vrn-B1a nor Vrn-B1c. An increased frequency of occurrence of the Vrn-B1c allele was revealed for Western Siberia (see Table 3, Fig. 3, b) and Vrn-B1a (see Fig. 3, a) for Eastern Siberia.

**Fig. 4.**
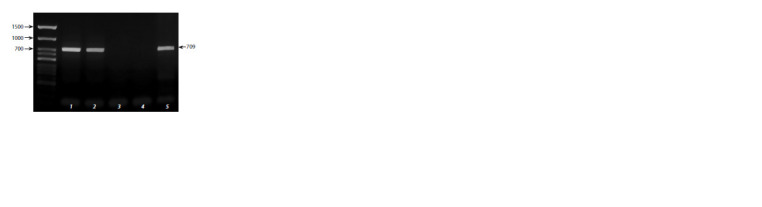
The gel electrophoresis of the PCR fragments contained the f irst intron of the Vrn-B1 gene in the tested cultivars. 1 – Baganochka; 2 – ANK; 3 – k-39218 T. aestivum var. lutinf latum Zhuk.; 4 – k-30234 T. araraticum Jakubz.; 5 – Barnaulskaya 32. The arrow marks the resulted fragments of 709 bps in size.

Fig. 4 presents gel electrophoresis of the PCR fragments contained the first intron of the Vrn-B1 gene in the tested cultivars. The amplification fragment of 709 bps marks the presence of the Vrn-B1a allele controlling a cultivar spring growth habit.

## Discussion

Morphotype. Plant breeders are commonly interested in complex analysis of the phenotypes of produced cultivars. For that reason, it was important for us to consider the setting significant volume accessions from 98 cultivars released in terms of morphotype difference between the cultivars from Western and Eastern Siberia and their dynamics during the last 100 years of scientific breeding during 1929–2021 (4 Preliminary zoning of agricultural crops in the State Breeding Test System and Protection was carried out in 1924 (Guidelines..., 1928).). For the purposes of the present study, a morphotype, i. e., the approbation (classification) traits of a common wheat cultivar, had been divided into two groups such as 1) awnedness/ awnedlessness and spike color that are known to affect the earliness (Pisarev, 1925); 2) grain color as a neutral parameter for there are no mentions about its effect in the publications. The results of data analysis presented at Table 3 and Fig. 2, b allow to conclude that the maximum contribution in the PC1 came from the region (both Eastern and Western Siberia), awnedness (r = 0.859) and spike color (r = 0.893). Another conclusion is that red spike ( ferrugineum, milturum) and awned ( ferrugineum, erythrospermum) varieties prevailed in Eastern Siberia (r = 0.863).

While other main morphotypes were distributed more or less equally with a little prevalence of white spike and awnedless varieties (lutescens) in Western Siberia (r = 0.321). The main contribution in the PC1 came from the spike color and its awnedness. Our study has confirmed the multiple conclusions made about the prevalence of red spike ( ferrugineum, milturum) and awned ( ferrugineum, erythrospermum) varieties in Eastern Siberia, and of whitespike awnedless varieties (lutescens) in Western Siberia (see Fig. 2, a, b)

1000-grain weight. This trait is correlated with yield (Melnikova et al., 2020) and milling quality parameters (Pototskaya et al., 2019). Our study demonstrated that the cultivars of Eastern Siberia were more small-grained than those from Western Siberia (see Table 3, Fig. 2, c). Earlier it had been demonstrated that the cultivars grown in the North Kazakhstan were also more small-grain than those farmed in Western Siberia (Moskalenko, 2007), so a conclu- 4 Preliminary zoning of agricultural crops in the State Breeding Test System and Protection was carried out in 1924 (Guidelines..., 1928). sion can be made that more continental climatic conditions determine the small size of the grain. The trait “1000-grain weight” is correlated with the duration of vegetation period (r = 0.410).

Estimating the correlation between the region and the two the most important traits such as earliness and 1000-grain weight using the methods of multivariate statistics brought us to the conclusion that the first was related to the regional component and the second – to that of polymorphism of trait “1000-grain weight”.

Vrn genes. The results obtained in our study make it possible to say that spring growth habit-related polymorphism in the Siberian cultivars of common wheat is supported only by the alleles of two dominant Vrn genes: Vrn-A1 and Vrn- B1 (see Supplementary Material). Furthermore, in 95 % of studied local cultivars and landraces of Siberia (Goncharov, Shitova, 1999) and Tuva (Moiseeva, Goncharov, 2007) this polymorphism is determined by two dominant Vrn genes.

For the dominant Vrn-A1 gene, the presence of two alleles, Vrn-A1a and Vrn-Ab, was shown (Lysenko et al., 2014; Efremova et al., 2016; etc.). The last allele is rather rare in Siberian cultivars and in our study was found only in 2 % of all tested ones (see Supplementary Material). It is possible that another allele of this gene can be found in the cultivars of North Kazakhstan, a territory that borders Western Siberia (Koval, Goncharov, 1998).

At the same time, allelism at the VRN-B1 locus is widespread in the Siberian cultivars (Shcherban et al., 2012a). Herewith, the Vrn-B1с allele prevails in the cultivars with monogenic spring growth habit control from Western Siberia and Northern Kazakhstan (Shcherban et al., 2012b). The same authors consider that in absence of the epistatic effects of the dominant Vrn-A1 gene, this allele provokes earlier earing if compared to Vrn-B1a allele, which enables these plants to evade first autumn frosts. However, the cases of monogenic control in Western Siberian cultivars are quite rare: in the past 70 years only two such spring cultivars have been registered that comprises 2 % of the spring cultivar sets in Siberia. At what, both cultivars were produced in today’s Omsk Agricultural Research Center.

Increasing the number of tested commercial cultivars and applying the principal component method for processing the genogeographic data has enabled us to demonstrate the non-random distribution of the dominant Vrn-B1 alleles in the cultivars of Western and Eastern Siberia (see Fig. 3, а and b). And, if earlier it was concluded that it is selective only for late-ripening varieties of Western Siberia and Northern Kazakhstan with monogenic control by Vrn-B1 (Shcherban et al., 2012a, b), our study detected the digenic control of highly-frequent Vrn-B1с (Western Siberia) and Vrn-B1а (Eastern Siberia) alleles (see Supplementary Material, Fig. 3, а, b). These findings make it possible to conclude that such digenic control is an optimal combination for the climatic conditions of Western and Eastern Siberia and confirms a possible breedability of multiple Vrn-B1 alleles in the cultivars with digenic control.

Using published data also allowed us to compare the frequencies of the genotypes with different dominant Vrn-1 alleles in the cultivars of Siberia and its neighboring regions (Moiseeva, Goncharov, 2007; Efremova et al., 2016). The analysis demonstrated that none of the considered cultivars had the dominant Vrn-D1 gene typical for the neighboring regions of China and Central Asia. This observation may be an additional argument in favor of the European origin of the modern Siberian cultivars.

The only cultivar to have trigenic spring growth habit control was cv. Tulun 15 (Lysenko et al., 2014), which is probably too many for a Siberian cultivar. Indirectly, this has been confirmed by the results of investigation into ultraearly- ripening common wheat lines Rico, Rimax, Fori, Rifor from the north-west of Russia, whose spring growth habit was controlled by Vrn-A1, Vrn-B1, Vrn-D1 and Ppd-D1а genes (Rigin et al., 2019, 2021a). At the same time, Tulun 15 has a different dominant Vrn gene than theirs, namely, Vrn- B3 typical of Chinese wheat (Zhang et al., 2008). Its other dominant gene is Ppd-D1a (Berezhnaya et al., 2021) that is alien not only for Siberia but for Russia as well (Balashova, Fait, 2021). Note the Prilenskaya cultivar zoned in Siberia also has a dominant Ppd-D1a gene (Lysenko et al., 2014). However, the photoperiodic sensitivity of Siberian cultivars was beyond the scope of our investigation.

We believe that increasing the spring growth habit-related polymorphism of common wheats requires either introgressing the dominant Vrn alleles from their wild relatives (Goncharov, Chikida, 1995; Goncharov, 1998) or using the rare alleles available in the gene pool of commercial cultivars (Stelmakh, Avsenin, 1996; Koval, Goncharov, 1998) that are understudied and have rarely been used in breeding. Whether the dominant Vrn-B3 gene can be used in breeding remains an open issue that requires further investigation. For the time being, only the Vrn-A1 and Vrn-B1 have shown the multiple alleles affecting the earing period. It is noteworthy that the donor of the Au T. urartu Thum. ex Gandil. genome provides no new mutations for spring common wheat (Golovnina et al., 2010) as well as using T. monococcum L. (Gonchrov et al., 2007) will never prove worthy.

Fig. 4 presents gel electrophoresis of the PCR fragments contained the first intron of the Vrn1B gene in the tested cultivars demonstrates the diagnostic product of the Vrn-B1 gene. It is 709 bps in size and was obtained from the Barnaulskaya 32 (Ozimka) spring cultivar. It is said to be produced by transformation of a winter cultivar into a spring one (Catalog…, 2009). In the sequence presents the standard deletion characteristic for other Siberian cultivars. Probably Barnaulskaya 32 is not a result of transformation, so its mutation cannot be used to extend Vrn polymorphism.

Cultivar zoning time. Changes of cultivar morphotypes in dynamic is another interesting topic and a subject of vivid discussions among breeders (Goncharov N.P., Goncharov P.L., 2018). Until the 1990s, breeding for awned and red-spike cultivars was clearly maintained in Eastern Siberia (see Fig. 2, a) (Catalog..., 2009). Since 1990 this tendency has changed due to disbanding a number of scientific and research facilities that carried out planned breeding and provided scientific supervision (Goncharov, Kosolapov, 2021).

It is a well-known fact that before starting to breed a new cultivar, a breeder should set strategic goals and find the ways of their achievement taking into account that in 15–20 years the requirements for the cultivar can change drastically due to changes in the economic situation, farming and processing techniques. However, within a properly organized breeding process, producing a new cultivar should not be a problem for it is what happens on regular basis and new cultivars being the products of the breeding programs started earlier are regularly sent to the State Commission for Breeding Test System and Protection. The only problem with this approach is to carefully preserve succession that refers both to the people and plants. In this respect, it would be interesting to investigate what germplasm materials had entered Siberian fields since the interregional program of the Kazakhstan-Siberian network for wheat improvement (KASIB) was launched (Kuz’min et al., 2019).

It goes without saying that cultivar replacement is crucial for crop farming in the Siberian Federal District. However, a cultivar must be cultivated as long as it can provide a sufficient yield of high quality.

Duration of vegetation period. Comparing the obtained data against the results for local cultivars highlighted the absence of sufficient changes in the frequencies of Vrn genes in the commercial cultivars of Siberian common wheat at least during the last 100 years (see Supplementary Material). The first preliminary zoning of wheat cultivars in our country was performed by V.V. Talanov in 1924 (Guidelines…, 1928), while the first Soviet Unione State Cultivar Zoning Register was produced only in 1929.

Duration of vegetation period is one of the main breeding parameters to characterize a cultivar or a sample in terms of their ripeness (from early- to late-ripening thorough middleearly, middle-ripening, middle-late, etc.) (Goncharov N.P., Goncharov P.L., 2018). This scale varies is for different species, but earliness and lateness remain the most expressed characteristics of any agricultural species. The results obtained in our study and presented in Table 3 do not permit to make firm conclusions since the contributions of traits into their own vectors and dispersions included in the corresponding principal components were too small. Moreover, such traits as red spikes (r = 0.893) and awnedness (r = 0.859) were those that correlated with the primary component, i. e., they had smaller joint duration of vegetation period.

Another important thing was that the earliness trait did not include a regional component (see Table 3). So, despite the fact that a number of cultivars were zoned as in Eastern as in Western Siberia, their percentage was rather small even in the recent years when one started to zone wheat cultivars not by regions but by bigger federal districts.

When producing new cultivar, breeders proceed from the concept of matching the duration of vegetation period to the conditions of the proposed farming area. The retrospective

analysis of the most perspective trends of common wheat breeding demonstrated that the earliness/lateness of modern cultivars had no longer been related to a region but rather to an ecological zone (taiga, subtaiga, forest-steppe and steppe), which raises a question of its latitudinal/longitudinal components that have never been previously studied (Goncharov, Rechkin, 1993; Rechkin, Goncharov, 1993), for geographic sowing defiantly showed the non-latitudinal character of the trait expression (Goncharov, Rechkin, 1993). At the same time, N.I. Vavilov (1928) and E.S. Kuznetsova (1929) insisted on having two groups of plants for geographical sowing: the first is to limit the seedling – ripening period from the South to North, and the second – to extend this period. Today, only two spring common wheat cultivars from Siberia have a Ppd-D1a gene (Lysenko et al., 2014; Berezhnaya et al., 2021). The absence of a close relationship (correlation) between the duration of vegetation period in spring common wheat and them yield has been repeatedly shown (Vedrov, Chalipsky, 2009; Piskarev et al., 2018).

It is also noteworthy that the accumulated perennial data has enabled us to see the retrospective that is crucial considering the reduced level of scientific supervision of crop research both in Siberia and Russia.

## Conclusion

Breeding for earliness is one of the important directions of spring wheat breeding in Siberia. The accumulated perennial data has made it possible to apply the methods of multivariate statistics to extract the meaningful insights they contain. The simplicity and representability of the approach make it a useful tool for decision taking when it comes to including a new cultivar into the “State Register for Selection Achievements Admitted for Usage (National List)”.

The present study investigating the geographical distribution of dominant Vrn genes has allowed us to estimate the advantages of the cultivars with certain alleles of those genes for specific territories of Siberia. It has been found that the digenic spring growth habit control is an optimum solution for the harsh climatic conditions of both Western and Eastern Siberia. The performed retrospective analysis has made it possible to indicate the most perspective breeding trands and revealed that the earliness/lateness trait of many modern spring common wheat cultivars no longer regionally related to either Western or Eastern Siberia. Nevertheless, the Eastern cultivars mainly have the Vrn-B1а allele, and the Western one – Vrn-B1с allele

## Conflict of interest

The authors declare no conflict of interest.
